# Accurate detection of low prevalence *AKT1* E17K mutation in tissue or plasma from advanced cancer patients

**DOI:** 10.1371/journal.pone.0175779

**Published:** 2017-05-04

**Authors:** Elza C. de Bruin, Jessica L. Whiteley, Claire Corcoran, Pauline M. Kirk, Jayne C. Fox, Javier Armisen, Justin P. O. Lindemann, Gaia Schiavon, Helen J. Ambrose, Alexander Kohlmann

**Affiliations:** 1 Personalised Healthcare and Biomarkers, Innovative Medicines and Early Development, AstraZeneca, Cambridge, United Kingdom; 2 Horizon Discovery Ltd, Cambridge, United Kingdom; 3 Early Clinical Development, Innovative Medicines and Early Development, AstraZeneca, Cambridge, United Kingdom; 4 Translational Science, Innovative Medicines and Early Development, AstraZeneca, Cambridge, United Kingdom; Rutgers, the State Univesity of New Jersey, UNITED STATES

## Abstract

Personalized healthcare relies on accurate companion diagnostic assays that enable the most appropriate treatment decision for cancer patients. Extensive assay validation prior to use in a clinical setting is essential for providing a reliable test result. This poses a challenge for low prevalence mutations with limited availability of appropriate clinical samples harboring the mutation. To enable prospective screening for the low prevalence *AKT1* E17K mutation, we have developed and validated a competitive allele-specific TaqMan^®^ PCR (castPCR™) assay for mutation detection in formalin-fixed paraffin-embedded (FFPE) tumor tissue. Analysis parameters of the castPCR™ assay were established using an FFPE DNA reference standard and its analytical performance was assessed using 338 breast cancer and gynecological cancer FFPE samples. With recent technical advances for minimally invasive mutation detection in circulating tumor DNA (ctDNA), we subsequently also evaluated the OncoBEAM™ assay to enable plasma specimens as additional diagnostic opportunity for *AKT1* E17K mutation testing. The analysis performance of the OncoBEAM™ test was evaluated using a novel *AKT1* E17K ctDNA reference standard consisting of sheared genomic DNA spiked into human plasma. Both assays are employed at centralized testing laboratories operating according to quality standards for prospective identification of the *AKT1* E17K mutation in ER+ breast cancer patients in the context of a clinical trial evaluating the AKT inhibitor AZD5363 in combination with endocrine (fulvestrant) therapy.

## Introduction

Oncology is at the frontline of personalized healthcare, utilizing the molecular profile of an individual’s cancer genome to tailor treatments to individual patients. This personalized approach depends on adequate diagnostic tests that enable prospective selection of patients. Diagnostic assays used for patient screening require extensive validation to ensure reliable and reproducible performance [1, 2]. In signal-searching clinical trials, diagnostic assays support rational drug development, and enhance our understanding of why experimental targeted therapies succeed or fail [3].

AZD5363 is a potent pan-AKT kinase inhibitor that has been shown to inhibit the growth of a range of human tumor xenografts in breast cancer models, and to produce significant tumor regression when combined with docetaxel or fulvestrant in breast cancer xenografts [4, 5]. In Phase 1 studies, AZD5363 monotherapy showed clinical activity in various heavily pre-treated solid tumors harboring the gain-of-function *AKT1* E17K mutation, including ER+ breast, cervical and ovarian cancer, and lung adenocarcinoma [6, 7], and is being tested in multiple basket trials and in combination with chemo- or hormonal therapy in various tumour types.

The p.E17K mutation is a single amino acid mutation in the pleckstrin homology (PH) domain of the AKT1 protein, a member of the PI3K/AKT/mTOR signaling pathway regulating cell cycle progression and proliferation [8]. According to the Catalogue of Somatic Mutations in Cancer (COSMIC) database (version 62), the *AKT1* E17K hotspot mutation is present in approximately 4% of breast tumors, 2.5% of endometrial cancers and 0.3% of ovarian cancers [9]. Despite its low prevalence, *AKT1* E17K has been reported to be an oncogenic driver mutation in breast cancer and other solid cancers [10–12]. A diagnostic assay for the *AKT1* E17K mutation will facilitate assessment of clinical activity of the pan-AKT inhibitor AZD5363 in advanced cancer patients whose tumor harbors this oncogenic mutation. Available assays are however for research use only, and have not been extensively validated using clinical samples.

Here, we describe the rationale and steps taken to deliver an analytically validated castPCR™ assay to detect the low prevalence *AKT1* E17K mutation in FFPE breast and gynecological tumor tissue samples. In addition, we present an analytical assessment of the OncoBEAM™ AKT1 assay using a newly developed ctDNA reference standard. This assay enables contemporary identification of the *AKT1* E17K mutation in advanced breast cancer patients using a plasma sample. Both assays are now employed for prospective mutation testing at centralized laboratories in a Phase 1 clinical study.

## Materials and methods

### FFPE tissue samples

In total, 388 FFPE samples were obtained from four commercial providers: Asterand Bioscience (Royston, UK), Manchester Cancer Research Centre Biobank (Manchester, UK), Wales Cancer Bank (Cardiff, UK) and Tissue Solutions (Glasgow, UK). A total of 195 FFPE tissue blocks were derived from ER+ breast tumors, 143 samples from gynecological (ovarian, endometrial and cervical) tumors, and 50 samples from normal breast tissue. All procedures were carried out in accordance with the Helsinki Declaration (1964, amended in 1975, 1983, 1989, 1996 and 2000) of the World Medical Association and all patient samples submitted for analysis were done so with the full informed consent of the donors: www.asterandbio.com/company/ethics, www.mcrc.manchester.ac.uk/Biobank/Ethics-and-Licensing, www.walescancerbank.com, and www.tissue-solutions.com/ethics

### Pathology review of tissue samples

The tumor samples were assessed by a pathologist at AstraZeneca to estimate the tumor cell content of the tissue samples prior to DNA extraction. Tumor cell content ranged widely across the samples, from <10% to >90%, with the majority (284/338; 84%) of samples containing at least 50% tumor material.

### DNA extraction from FFPE human tumor samples

Genomic DNA (gDNA) was extracted from FFPE tissue specimens using the Roche **cobas**^®^ DNA Sample Preparation Kit (Roche Molecular Systems, Pleasanton, CA). One or two 5 μm mounted sections were used for each tissue extraction according to the manufacturer protocol. DNA was quantified using a NanoDrop spectrophotometer (Thermo Fisher Scientific, Waltham, MA).

### Healthy donor plasma samples

For the evaluation of the OncoBEAM™ AKT1 assay, twenty 5 mL frozen plasma samples from 20 healthy female donors were purchased from Tissue Solutions (Glasgow, UK) with appropriate informed consent under UK Human Tissue Authority Licensing. Blood was collected in K3 EDTA vacutainers, and centrifuged 10 minutes at 2000 x g at 4°C. The plasma was centrifuged a second time for 10 minutes at 2,600 x g, aliquoted into 2 mL cryovials and stored at –80°C. One 2 mL aliquot of each donor was used for the OncoBEAM™ AKT1 assay.

For the development of the *AKT1* E17K ctDNA reference standard, plasma was purchased from The Medicines Evaluation Unit (MEU; Manchester, UK). Blood was collected from 10 healthy donors (60 mL per donor) in K3 EDTA vacutainers and processed as described above. Equal amounts of plasma from each donor was combined to obtain 250 mL pooled plasma. The pooled plasma was subsequently aliquoted into ten 25 mL aliquots, which were stored at –80°C prior to use for preparation of the ctDNA reference standard as described below.

### castPCR™ assay reagents and instrumentation

The competitive allele-specific TaqMan^®^ PCR (castPCR™) assay (Thermo Fisher Scientific) was performed using the ABI PRISM® 7900HT Sequence Detection System (Thermo Fisher Scientific). The instrument was operated in absolute quantification standard curve mode according to the manufacturer’s instructions. Amplification conditions used comprised one denaturation cycle (95°C, 10 minutes), five pre-amplification cycles (92°C for 15 seconds, 58°C for 60 seconds) and 40 PCR cycles for denaturation, annealing and product extension (92°C for 15 seconds, 60°C for 60 seconds). DNA was diluted to 5 ng/μL, and 4 μL (20 ng) DNA was included in each amplification reaction. The following reagents were used: TaqMan^®^ Mutation Detection Assay AKT1_33765_mu, TaqMan^®^ Reference Detection Assay AKT1_rf, TaqMan^®^ Genotyping Master Mix 2x and the Internal Positive Control (IPC) Kit (Thermo Fisher Scientific). The IPC comprised a synthetic DNA sequence not homologous to a human genomic sequence. Primers and a fluorescent signal detection probe from the IPC kit, which were specific for the IPC amplification product, provided an internal control for PCR failure. Two negative controls comprising all reagents (including IPC but excluding other sources of template DNA, i.e. with 4 μL distilled nuclease-free water replacing the template) were prepared and included each time the assay was performed as well as positive controls comprising *AKT1* E17K cell line admixtures. Amplification reactions were prepared in a standard 96-well plate format. Each operation of the assay, including plate preparation and amplification, yielded mutation results for 48 tumor samples in less than 5 hours.

### *AKT1* E17K cell line DNA and FFPE DNA reference standards

For the high quality genomic DNA reference standard, the KU19-19 cell line, heterozygous for the *AKT1* E17K mutation, was purchased from DSMZ (Braunschweig, Germany) and grown at the AstraZeneca cell bank according to the recommended conditions. Following DNA extraction as described above for human tissue samples, DNA was quantified using a NanoDrop spectrophotometer and mixed with wild-type human genomic DNA (Roche Diagnostics, Mannheim, Germany) to obtain 1%, 2%, 5% or 10% mutant DNA copies in 2x10^5^ wild-type DNA copies per mL 0.1 x TE, assuming 3.3 pg per genomic equivalent (S1 Table).

For the FFPE DNA reference standard, an *AKT1* E17K heterozygous cell line and corresponding *AKT1* wild-type cell line fixed in formalin and embedded in paraffin to mimic FFPE tissue samples were purchased from Horizon Diagnostics (Cambridge, UK; catalogue numbers HD167 and HD172). Following DNA extraction and quantification as described for human tissue samples, the *AKT1* E17K mutant and wild-type DNA was blended to obtain 1%, 2%, 5% or 10% mutant allele frequencies in a background of 2x10^5^ wild-type DNA copies per mL 0.1 x TE, assuming 3.3pg per genomic equivalent (S1 Table).

### *AKT1* E17K ctDNA reference standards

The ctDNA reference standard was developed by Horizon Discovery (Cambridge, UK) using an *AKT1* E17K heterozygous cell line and corresponding *AKT1* wild-type cell line (catalogue numbers HD658 and HD659). Genomic DNA was extracted from both cell lines using the Maxwell 16 DNA purification Kit (Promega, Madison, WI) according to manufacturer’s instructions. Extracted DNA was quantified using a NanoDrop spectrophotometer, diluted to 50 ng/μl, and sheared to an average peak size of 170 bp using the Covaris sonicator (Covaris, Woburn, MA). Fragment size was measured by the Agilent TapeStation instrument using D1000 reagents (Agilent Technologies, Santa Clara, CA). Sheared DNA was quantified using Qubit® dsDNA BR Assay kit (Thermo Fisher Scientific), according to manufacturer’s instructions. Sheared DNA of both cell lines was blended to obtain 0.05%, 0.5%, 1%, 2% and 5% mutant allele frequencies, assuming 3.3 pg per genomic equivalent. The admixtures were spiked into pooled healthy donor plasma (described above) to obtain 6 x 10^4^ *AKT1* copies per mL plasma, taking into consideration the low presence of wild-type *AKT1* in the plasma (S1 Table).

### Digital droplet PCR to detect the *AKT1* c.49G>A (p.E17K) mutation

The *AKT1* E17K (c.49G>A) mutant allele frequencies in the ctDNA reference standard samples were validated using a digital droplet polymerase chain reaction (ddPCR) *AKT1* assay on the QX100™ ddPCR system (BioRad, Hercules, CA), using 50 ng DNA per reaction. The ddPCR conditions were: one denaturation cycle (95°C, 10 minutes), 40 PCR cycles for denaturation, annealing and product extension (94°C for 30 seconds, 61°C for 60 seconds) and one step for enzyme deactivation (98°C, 10 minutes). The following primers and probes were used for the reaction: Forward Primer Sequence: GAGTGTGCGTGGCTCTCA; Reverse Primer Sequence: GCCATCATTCTTGAGGAGGAAGTAG; Reporter VIC Sequence: CAGGTCTTGATGTACTCCCCTA; Reporter FAM Sequence AGGTCTTGATGTACTTCCCTA. Each plate included appropriate negative controls, including no-template controls or DNA only but no ddPCR mix or probe controls. Wild-type *AKT1* DNA controls were included to assess background detection levels of the *AKT1* E17K mutation.

### *AKT1* E17K mutation analyses at external service laboratories

Genomic DNA extracted from all clinical FFPE samples was supplied to Sequenom® GmbH (Hamburg, Germany) for mutation analysis using the iPlex Matrix Assisted Laser Desorption/Ionisation Time of Flight Mass Spectrometry (MALDI-TOF MS) method [13] and a custom panel covering 49 mutations, including the *AKT1* E17K mutation. At the time of the analysis, at least 10% of the analysed *AKT1* copies should contain the E17K mutation in order to classify an FFPE sample as ‘mutation positive’.

The BEAMing-based mutation confirmatory analysis on 10 FFPE samples was performed at the Good Clinical Practise (GCP)-certified service laboratory of Sysmex Inostics GmbH (Hamburg, Germany). Genomic DNA extracted from 6 breast and 4 gynecological cancer FFPE samples for which the *AKT1* E17K mutation was detected by castPCR™, was supplied to Sysmex Inostics GmbH for analysis using BEAMing technology. At the time of the analysis, at least 1% of the analysed *AKT1* copies should contain the E17K mutation in order to classify an FFPE sample as ‘mutation positive’.

The BEAMing-based mutation analyses on ctDNA samples was performed at the Sysmex Inostics Service Laboratory in Baltimore (MA, USA), which is certified within the United States Federal Government's clinical laboratory oversight program (CLIA). Plasma samples were supplied to Sysmex Inostics for DNA extraction and *AKT1* E17K mutation analysis using the OncoBEAM™ assay. At the time of the analysis, at least 0.02% of the analysed *AKT1* copies should contain the E17K mutation in order to classify a plasma sample as ‘mutation positive’.

## Results

### Establishment castPCR™ analysis parameters for *AKT1* E17K mutation detection in FFPE samples

**Fig 1:**
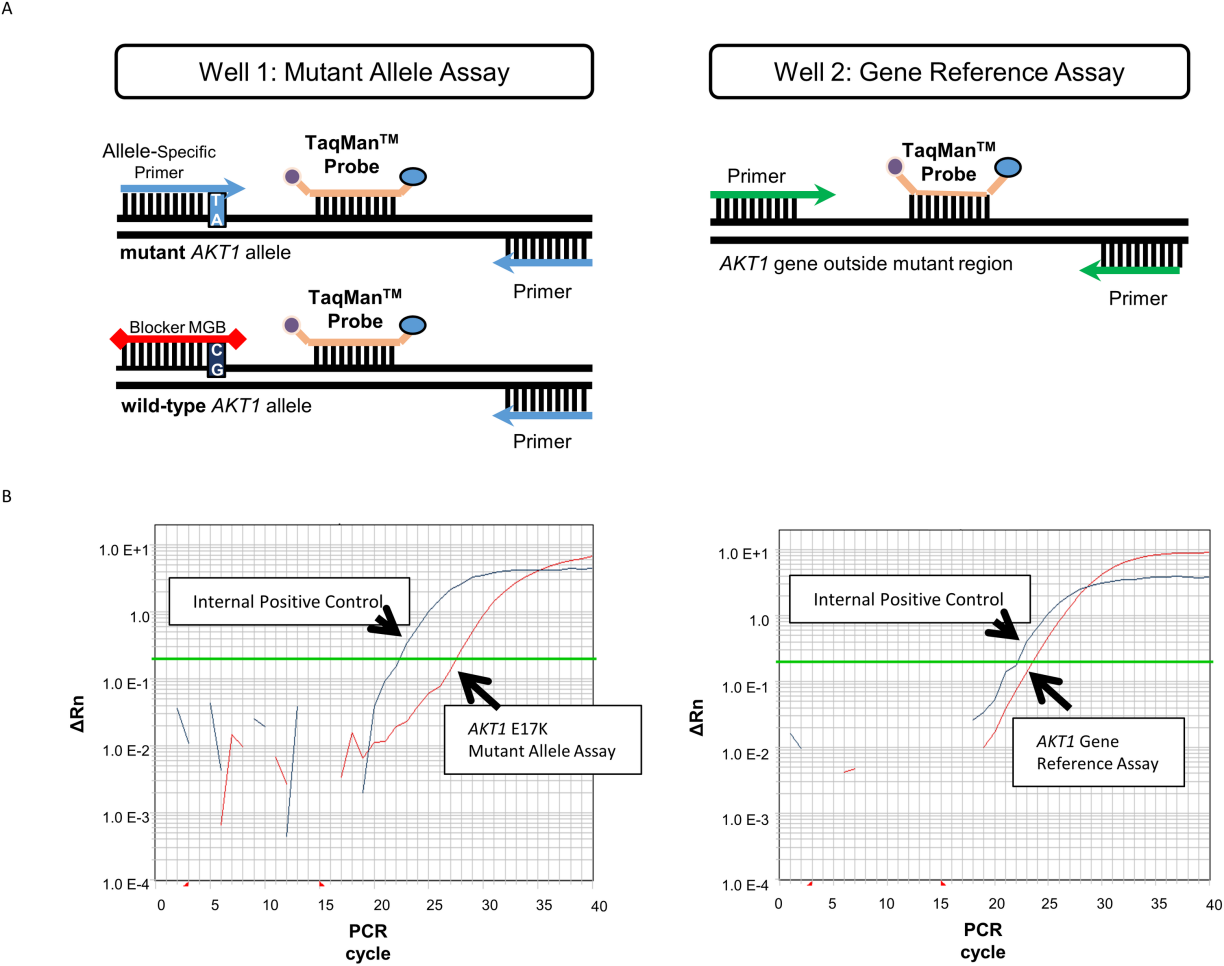
Schematic representation of the CastPCR^TM^ assay. (A) Two independent amplification reactions are required for mutation detection: the Mutant Allele Assay, in which the mutant allele is amplified via the mutation-specific primer whilst amplification of the wild-type DNA is suppressed by the blocker containing the minor groove binder (MGB) (left), and the Gene Reference Assay, in which a DNA region located within the same gene but outside the mutant region is amplified (right). Both assays include an internal positive control (IPC) to control for PCR failure. (B) Example of Amplification Plots from a breast cancer sample analysis. The first curve to cross the signal threshold line (green line) represents the signal generated by the IPC reaction (blue line). The second curve (red line) represents the signal generated by the Mutant Allele Assay (left) or Gene Reference Assay (right). In this example, the Ct values for the Gene Reference Assay and the Mutant Allele Assay were 23.5 and 27.5 respectively. The resulting ΔCt is 4, indicating that the castPCR™ assay detected the *AKT1* E17K mutation in this sample.

A literature-based review identified the castPCR™ assay, a modified real-time PCR approach combining TaqMan^®^ quantitative PCR, as a potential suitable method for prospective *AKT1* E17K mutation detection in a clinical study. The castPCR™ method is an 2-well PCR-based assay (Fig 1) and considered sensitive and specific as it selectively amplifies the mutant DNA sequence of interest (the *AKT1* E17K mutation in this study, i.e. nucleotide A at position 49 in the *AKT1* gene), by suppressing amplification from the wild-type DNA sequence (nucleotide G at position 49 in the *AKT1* gene). Since the assay had not been validated using FFPE samples, we first set out to establish the threshold values for the mutation assay Ct, reference assay Ct, and ΔCt for *AKT1* E17K mutation detection in FFPE samples using the *AKT1* E17K FFPE DNA reference standards and included DNA from 10 *AKT1* wild-type FFPE breast tumors as a negative control. Each tumor specimen was analyzed in triplicate, whilst the FFPE DNA reference standards were assayed 11 times (Table 1). For the wild-type DNA from the FFPE breast tumors, a ΔCt between the reference and mutation assay of >8.1 was maintained between 18 and 30 amplification cycles. The mutant *AKT1* containing reference standard generated a detectable signal such that the ΔCt was <8.1 cycles down to 5–10% *AKT1* E17K sequence (Table 1). Therefore, a ΔCt cut-off of 8.1 was selected as the maximum ΔCt acceptable for the assay result to be considered ‘mutation detected’. However, the ΔCt values observed in the FFPE reference standard varied more than those observed using the KU19-19 cell line DNA reference standard (Table 1). It was therefore established that a sample should be repeated in duplicate to confirm the mutation status if the ΔCt value was <8.1 but ≥7, in order to minimize false-positive mutation detection. Furthermore, if the reference assay failed to produce a detectable PCR product within the 18- to 30-cycle window, the assay must be considered to have failed as the relationship between the amplification signals was not reliable. Also, if the internal positive control (IPC) reaction, which controls for optimal amplification, failed to produce a signal within 30 cycles, the assay must be considered to have failed. In those cases, a repeat assay with adjusted amount of input DNA should be performed. A summary of the data interpretation rules is presented in Table 2.

**Table 1:** Individual and mean ΔCt results of two *AKT1* E17K DNA reference standards analyzed by castPCR™ to determine analysis parameters.

Sample type	*AKT1* E17K (%)	ΔCt												
		Run 1	Run 2	Run 3	Run 4	Run 5	Run 6	Run 7	Run 8	Run 9	Run 10	Run 11	Mean	St. dev
**High quality DNA reference standard** (KU19-19)	50	-4.98	-4.77	-4.16	-4.38	-4.33	-4.44	-4.41	-4.67	-4.28	-4.45	-4.39	-4.48	0.24
	10	-7.36	-7.08	-6.63	-6.71	-6.19	-6.50	-6.54	-6.63	-6.60	-6.70	-6.46	-6.67	0.31
	5	-8.02	-7.89	-7.57	-7.77	-7.55	-7.44	-7.60	-7.83	-7.76	-7.86	-7.99	-7.75	0.19
	2	-8.82	-8.35	-8.15	-9.30	-8.45	-8.62	-9.06	-8.02	-8.42	-8.91	-8.59	-8.61	0.39
	1	-9.98	-10.25	-9.82	-8.73	-8.88	-8.99	-8.87	-9.82	-9.66	-9.00	-9.37	-9.40	0.53
	0 (2x10^5^)	-	-	-15.53	-	-	-	-	-	-15.16	-	-15.45	-15.38	0.20
	0 (2x10^6^)	-	-18.81	-15.50	-17.43	-18.00	-16.77	-16.50	-16.41	-19.36	-15.12	-20.66	-17.46	1.76
**FFPE DNA reference standard** (HD167 and HD172)	50	–4.64	–4.43	–5.90	–4.58	–4.83	–4.86	–4.72	–4.64	–4.45	–4.91	–3.77	-4.70	0.50
	10	–8.13	–5.83	–7.11	–5.53	–6.50	–5.36	–8.32	–4.28	–4.56	–7.52	–6.53	-6.33	1.36
	5	–5.81	–9.50	–5.09	–9.81	–6.81	–10.65	–6.17	–5.50	–5.86	–6.40	–8.66	-7.30	1.97
	2	–9.62	–8.64	-	–6.68	-	–7.02	–4.71	–4.92	-	-	–5.77	-6.77	1.84
	1	-	–9.09	–9.27	-	–3.01	–6.39	-	-	–6.59	–5.74	-	-6.68	2.32
	0 (2x10^5^)	-	-	-	-	-	-	-	-	-	-	-	-	-
	0 (2x10^6^)	-	-	-	-	-9.16	-11.03	-	-	-	-	-	-10.10	1.32

A single reference standard sample was used for runs 1–11. The reference standards consisted of 2x10^5^ *AKT1* copies per mL; the negative control (0% *AKT1* E17K) was run in two different concentrations: 2x10^5^ and 2x10^6^ copies per mL. ‘-‘ indicates that no ΔCt could be determined as the Mutant Allele Assay did not produce a Ct value.

**Table 2:** Interpretation of castPCR™ *AKT1* E17K assay results.

Assay component	Ct value	ΔCt	Result interpretation	Recommendation
Reference assay	18–30	–	Reference assay pass	–
	<18	–	Reference assay fail	Repeat sample with less DNA
	>30 or no signal	–	Reference assay fail	Repeat sample with more DNA
Mutant assay Ct	>37	<8.1	Undetermined	Repeat sample in duplicate
	>37	>8.1	Mutation not detected	–
	<37	<7	Mutation detected	–
	<37	7–8.1	Mutation detected	Repeat sample in duplicate for confirmation
IPC Mutant and/or Reference assay	>30 or no signal	–	Assay fail	Repeat sample
NTC Mutant and/or Reference assay	>30 or no signal	–	Plate pass	–
	<30	–	Plate fail	Repeat whole plate

Ct, cycle threshold; ΔCt, difference in Ct values of Mutant Allele Assay minus Gene Reference Assay; IPC, internal positive control; NTC, no template control.

### Analytical validation of the castPCR™ assay on 195 FFPE breast tumors samples

To determine the accuracy of the established *AKT1* E17K castPCR™ assay cut-off values in FFPE from clinical samples, DNA derived from 195 FFPE breast cancer tumor samples with unknown mutation status was assayed by castPCR™. Six *AKT1* E17K mutation positive samples were identified in this cohort, a mutation detection rate of 3.1%. These results were compared with a second independent method, MALDI-TOF MS-based analysis at Sequenom GmbH. With the exception of a single *AKT1* E17K positive sample, all samples (194/195) yielded identical mutation status results. The discordant sample (50093A1) did actually reveal an *AKT1* E17K mutation by MALDI-TOF MS analysis, but the sample was classified as ‘no mutation detected’ as it fell below the assay’s detection threshold. To confirm the true mutation status of this sample, all six samples with a ‘mutation detected’ result by castPCR™ were also analyzed by the highly sensitive BEAMing method at Sysmex Inostics GmbH. BEAMing detected the *AKT1* E17K mutation in all six samples, including at low level in sample 50093A1 (Table 3), confirming no false-positive mutation detection by the castPCR™ assay.

**Table 3:** Comparison of *AKT1* mutation detection by castPCR™, MALDI-TOF MS and BEAMing in 6 *AKT1* E17K mutation positive breast cancer samples.

Sample Identifier	castPCR™	MALDI-TOF	BEAMing	Agreement	CastPCR ΔCt	MALDI-TOF MAF (%)	BEAMing MAF (%)
1148529B	Mutation detected	Mutation detected	Mutation detected	Concordant	4.1	74.1	42.0
1158495B	Mutation detected	Mutation detected	Mutation detected	Concordant	4.9	39.9	31.2
126607A2	Mutation detected	Mutation detected	Mutation detected	Concordant	5.1	39.2	22.5
1146714B	Mutation detected	Mutation detected	Mutation detected	Concordant	5.0	39.0	30.0
IOM-600-5T*	Mutation detected	Mutation detected	Mutation detected	Concordant	5.8	33.7	14.1
50093A1*	Mutation detected	No Mutation detected**	Mutation detected	Discordant	6.9	6.6	3.4

A different DNA preparation sample was used for each evaluated method. MAF; mutant allele frequency.

*For samples 50093A1 and IOM-600-5T, mean ΔCt values generated from repeat analyses by three different laboratory personnel are presented.

**Noted as ‘no mutation detected’ as MAF was below the level of detection for MALDI-TOF MS.

### Analytical validation of castPCR™ assay on 142 FFPE gynecological tumors samples

To assess whether the castPCR™ can reliably detect *AKT1* E17K mutations in gynecological tumors samples as well, an analytical validation was performed using 142 gynecological FFPE tumor-derived DNA samples with unknown AKT1 mutation status. For these samples, castPCR™, Sequenom^®^ MALDI-TOF MS and OncoBeam™ digital PCR identified the same four *AKT1* E17K positive samples (Table 4), a mutation detection rate of 2.8%. The remaining 138 samples were found to be *AKT1* E17K mutation negative for both castPCR™ and MALDI-TOF MS analyses. These data demonstrate that the castPCR™ was highly sensitive and specific when compared to Sequenom^®^ MS genotyping.

**Table 4:** Comparison of *AKT1* mutation detection data generated by castPCR™, MALDI-TOF MS and BEAMing in 4 *AKT1* E17K gynecological cancer samples.

Sample Identifier	castPCR™	MALDI-TOF	BEAMing	Agreement	CastPCR ΔCt	MALDI-TOF MAF (%)	BEAMing MAF (%)
F13/03213	Mutation detected	Mutation detected	Mutation detected	Concordant	4.2	94.5	82.0
PZQXHALX*	Mutation detected	Mutation detected	Mutation detected	Concordant	3.7	93.2	89.5
114816A2*	Mutation detected	Mutation detected	Mutation detected	Concordant	3.8	79.4	63.3
F13/03219	Mutation detected	Mutation detected	Mutation detected	Concordant	4.4	62.0	45.3

A different DNA preparation sample was used for each evaluated method. MAF; mutant allele frequency.

*For samples 114816A2 and PZQXHALX, mean ΔCt values generated from repeat analyses by three different laboratory personnel are presented.

### Inter- and intra-assay variation of the castPCR™ assay

**Fig 2:**
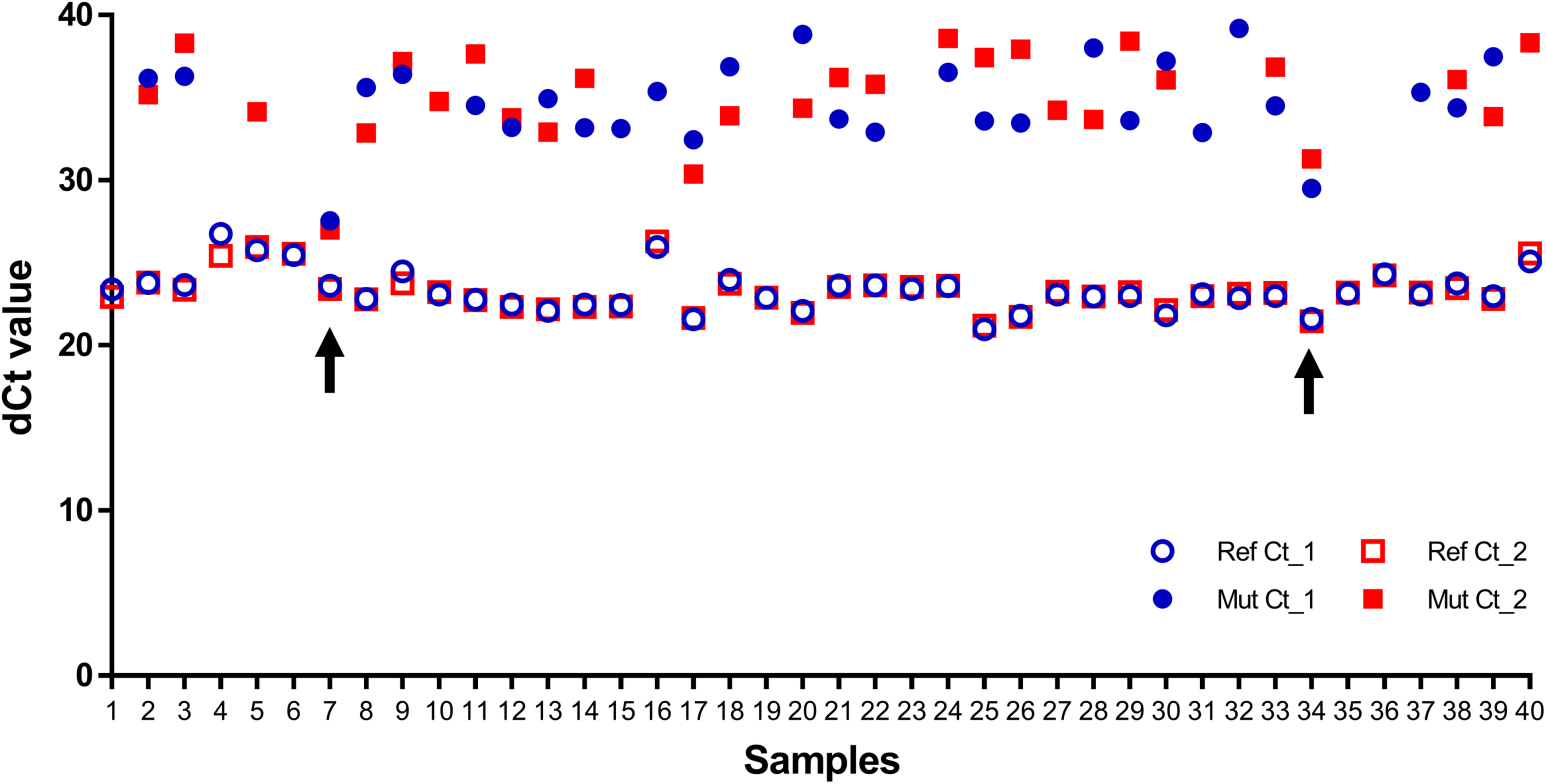
Ct values of duplicate assays showing intra-assay agreement. All samples were performed in duplicate, resulting in two Ct values for the Gene Reference Assay (open circle) and two Ct values for the Mutant Allele Assay (closed circle). The blue circles show the Ct values obtained in the first experiment, and the red circles show the Ct values obtained in the second experiment. Absence of a closed circle for a particular sample indicates that no Ct value was detected. The two samples in which the *AKT1* E17K mutation was detected (samples 7 and 41 with a ΔCt <8.1) are indicated with an arrow.

The intra-assay precision and inter-assay robustness were investigated to establish the level of performance variation introduced by operator-specific factors in order to assess whether the assay was suitable for transfer to external testing laboratories. To evaluate the intra-assay agreement, DNA extracted from 48 FFPE gynecological tumor samples with unknown mutation status was assessed in duplicate. All samples yielded 100% concordance from this duplicate analyses: 2 ‘mutation detected’ samples and 46 ‘no mutation detected’ samples (Fig 2).

To assess the inter-assay robustness, 48 breast tumor samples with unknown mutation status were assayed by three different laboratory personnel on different days, using the same instrument (ABI 7900) and the same batches of assay reagents. The mutation calls obtained by all three operators showed 100% concordance: 1 ‘mutation detected’ sample, 46 ‘no mutation detected’ samples, and 1 sample failed analysis by all three operators, suggesting that the DNA of this particular sample was inadequate.

Overall, these data indicate that the assay is reproducible between batches and between operators, and is sufficiently robust to transfer to external testing laboratories for routine diagnostic testing of FFPE breast and gynecological tumor tissue samples.

### Analytical validation of OncoBEAM™ assay using a novel *AKT1* ctDNA reference standard

**Fig 3:**
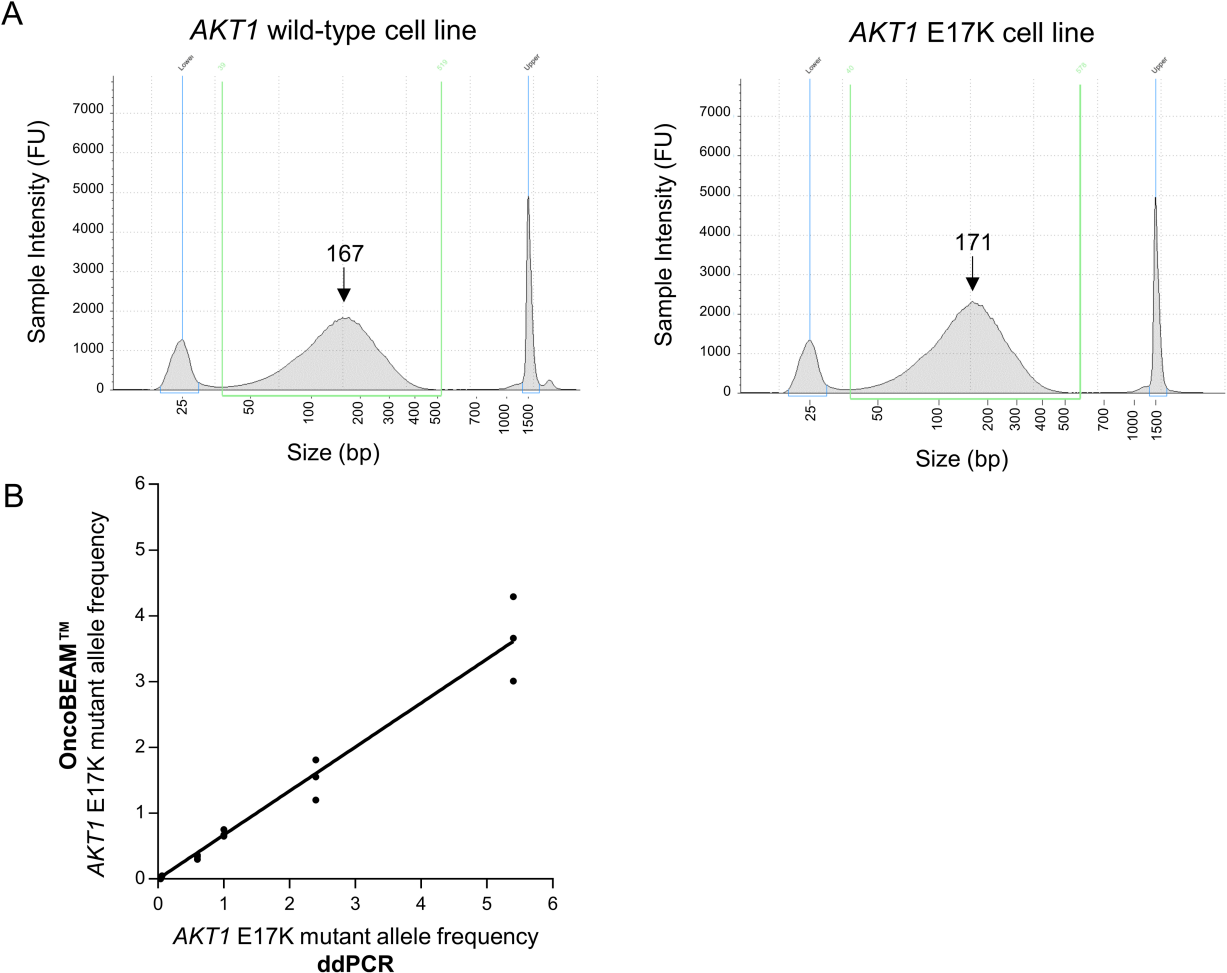
*AKT1* E17K ctDNA reference standard for OncoBEAM™ evaluation. (A) Representative TapeStation (D1000 screentape) graphs showing the DNA sizes from a wild-type *AKT1* (left) and an *AKT1* E17K harboring (right) cell line sample upon fragmentation by Covaris prior to spiking into plasma. The arrows demonstrate the average peak size. (B) Graph presenting the *AKT1* E17K mutant allele frequencies detected in the ctDNA reference standard samples analyzed in triplicate by the OncoBEAM™ assay (presented on the y-axis) and ddPCR assay (presented on the x-axis).

The highly sensitive OncoBEAM™ assay, a digital PCR assay with a detection cut-off of 0.02% mutant allele frequency for *AKT1* E17K, was available for plasma-based analysis in a CLIA-certified lab at Sysmex Inostics, Inc. (Baltimore, US) at the time of assay evaluation. However, we were limited in sourcing sufficient clinical plasma samples from metastatic breast cancer patients harboring an *AKT1* mutation due to the low prevalence of *AKT1* E17K mutations. Therefore, a novel ctDNA reference standard was developed with key characteristics of clinical plasma samples [14–17]. Genomic DNA from *AKT1* wild-type and matched *AKT1* E17K mutant cell lines was sheared to ~170bp (Fig 3A) and spiked into human plasma from healthy donors at 0.05%, 0.5%, 1%, 2% and 5% *AKT1* E17K mutant allele frequencies at 6x10^4^ copies/mL. This novel *AKT1* E17K ctDNA reference standard was assayed in triplicate in a blinded manner at the Sysmex Inostics CLIA laboratory to assess the performance of the OncoBEAM™ assay. The *AKT1* E17K mutation was accurately detected at all mutant allele frequencies in the ctDNA reference standard (Table 5). In addition, the OncoBEAM™ data demonstrated a statistically significant linear correlation with an orthogonal assay, digital droplet PCR (ddPCR) performed at Horizon Discovery (R^2^ = 0.9651, p<0.0001; Fig 3B).

**Table 5:** *AKT1* E17K mutation detection by OncoBeam™ digital PCR in ctDNA reference standard.

***ctDNA reference standard***	***ddPCR***	***OncoBEAM AKT1 E17K assay***	
expected MAF (%)	observed MAF (%) (n = 1)*	observed MAF (%) (n = 3)*	Mutation status
0	0.04**	0.004	no mutation detected
		0.003	no mutation detected
		0.003	no mutation detected
0.05	0.06	0.05	mutation detected
		0.03	mutation detected
		0.03	mutation detected
0.5	0.6	0.34	mutation detected
		0.30	mutation detected
		0.36	mutation detected
1.0	1.0	0.75	mutation detected
		0.67	mutation detected
		0.65	mutation detected
2.0	2.4	1.20	mutation detected
		1.55	mutation detected
		1.81	mutation detected
5.0	5.4	3.01	mutation detected
		3.66	mutation detected
		4.29	mutation detected

MAF; mutant allele frequency.

*One aliquot of each ctDNA reference sample was used in the ddPCR analysis with 3 wells per sample (or 6 wells for the 0.5% and 0.05% samples) to obtain >37,500 droplets. Three separate aliquots of the ctDNA reference samples were used for the OncoBEAM™ assay evaluation.

**The mutant copies detected in the wild-type *AKT1* sample were all double-positive droplets in the ddPCR analysis, likely due to non-specific binding of the probe or low level of polymerase-introduced errors during the amplification.

To assess the Limit of Blank (LoB) as well as the specificity of the OncoBEAM™ AKT1 assay, DNA from 20 healthy donor plasma samples was analysed. All samples were correctly classified as ‘no mutation detected’. In addition, the mean value detected in these 20 healthy donor plasma samples was 0.004%, with a Limit of Blank of 0.0098% (95% CI), which is more than 2-fold below the assay’s detection cut-off.

## Discussion

Here, we present data supporting two assays for prospective *AKT1* E17K mutation detection in clinical studies, the castPCR™ for FFPE tissue and the OncoBEAM™ for plasma-based mutation analysis. In order to select and develop an assay suitable to detect the *AKT1* E17K mutation in DNA from FFPE samples of ER+ breast tumors or tumors of gynecological origin (ovarian, endometrial and cervical) in a clinical trial, an assay with 100% specificity (i.e. no false positive test results) and good sensitivity (i.e. limited number of false-negative test results) was required. We therefore established stringent cut-off values for the castPCR™ assay (e.g. Ct and ΔCt cut-off values) to ensure accurate identification of *AKT1* E17K mutant patient population using this assay. The LoD of 5–10% for the castPCR™ assay developed in the current study is in line with the particular technology and instrument platform [18–20] and considered acceptable for FFPE-based mutation analysis. Other PCR-based diagnostic tests currently in use for patient selection have similar LoD (for example, 2–6% for various *KRAS* mutations in the **cobas®** KRAS Mutation Test, and 1–7% for various *EGFR* mutations in the therascreen® EGFR RGQ PCR test). In addition, the simplicity of the castPCR™ assay and wide availability of both the reagents and the platforms used for DNA extraction and PCR amplification facilitated transfer of the assay to testing laboratories operating according to quality standards appropriate for clinical trial delivery.^23^

Although the castPCR™ *AKT1* E17K assay performed well, the development of multi-gene panel approaches based on next-generation sequencing (NGS) for the selection of cancer patients for clinical studies is likely to replace single gene mutation assays for both clinical trial and therapy selection purposes [21]. However, regulatory and technical challenges as well as costs and test turnaround times associated with NGS platform-based diagnostic tests may limit the use of this technology for early phase clinical studies focusing on a limited number of gene mutations [22].

A challenge for analytical validation studies on assays developed for low prevalence mutations, such as the *AKT1* E17K mutation, is the limited availability of clinical samples harboring the mutation of interest. For the castPCR™ assay validation studies, we sourced >330 breast or gynecological tumor FFPE specimens. The mutation detection rate in this study, 6/195 (3.1%) and 2/142 (1.4%) for breast and gynecological samples respectively, was consistent with the low mutation prevalence reported [9, 12]. Although the absolute number of *AKT1* E17K mutant samples in the study was limited, the high concordance with orthogonal methods as well as the reproducibility in the intra- and inter-assay variability assessment supported the application of castPCR™ for clinical studies. Upon successful validation at partner CROs (data not reported), the castPCR™ assay is currently one of the methods accepted for prospective and retrospective *AKT1* E17K mutation testing of ER+ breast cancer samples for clinical trial NCT01226316.

Mutation analysis using circulating tumor DNA in plasma offers great benefit for patients with advanced disease. A blood sample represents the contemporary mutation status in contrast to an archival sample [23], is minimally invasive to obtain, and has a rapid test turnaround time [24, 25]. It also is not subject to the sampling bias caused by spatial heterogeneity of mutations within a tumour [26–28]. For metastatic breast cancer patients, it has been reported that the vast majority (>90%) of patients shed detectable levels of ctDNA into the blood circulation [29], indicating that plasma-based analysis could be a suitable approach for *AKT1* E17K mutation detection in this patient population. However, the amount of ctDNA in the plasma can be low [17, 29, 30], and highly sensitive technologies are required to detect tumour derived mutations. The OncoBEAM™ assay is a highly sensitive assay with a detection cut-off of 0.02% mutant allele frequency for plasma-based analysis, and was available in a CLIA-certified lab at Sysmex Inostics, Inc. (Baltimore, US) at the time of assay evaluation. In addition, the assay is compatible with the use of Streck® cell-free DNA Blood Collection Tubes (BCT), which preserve the sample for >5 days at ambient temperature [31], allowing sample processing at a central laboratory, minimizing variation in pre-analytical steps which could impact mutation detection [17]. Therefore, the OncoBEAM™ assay was selected for further validation studies to assess its suitability for prospective screening in a clinical study.

For the OncoBEAM™ *AKT1* plasma-based assay evaluations, insufficient clinical plasma samples harboring the *AKT1* E17K mutation were available. We therefore developed a novel ctDNA reference standards with key characteristics of clinical ctDNA, i.e. highly fragmented DNA, present at low copies in human plasma [14–17]. The OncoBEAM™ assay successfully detected all mutations in all technical replicates. However, the mutant allele frequencies reported by the OncoBEAM™ assay were slightly lower compared to those reported by the ddPCR assay (Fig 3B), which is likely due to technological differences, such as the pre-amplification step in the BEAMing technology, the inclusion versus exclusion of double-positive signals in ddPCR versus BEAMing assays, or the polymerase used to amplify the DNA [30, 32]. DNA polymerases may introduce errors during the amplification, and are particularly prone to G>A substitutions [33], which could potentially lead to false-positive test results in our study as the *AKT1* mutation of interest is the c.49G>A nucleotide change. Importantly, further evaluations on the risk of false-positive results using 20 healthy donor plasma samples demonstrated a background signal 5-fold below the detection threshold of the assay. Furthermore, the assay is currently in use for prospective and retrospective screening of ER+ metastatic breast cancer patients in the clinical study NCT01226316. In addition to a fast test turnaround time, initial results showing a 100% mutation detection concordance compared to tissue-based analysis performed at local sites, and a mutation detection rate of ~3% in plasma samples submitted for prospective screening to date.

In summary, this study has identified and validated a castPCR™ assay for detecting the *AKT1* E17K mutation in FFPE breast and gynecological tumor tissue samples, and subsequently confirmed the suitability of the OncoBEAM™ assay for *AKT1* E17K mutation detection in plasma samples of advanced metastatic breast cancer patients. Both methods are employed at central testing labs for prospective or retrospective identification of the *AKT1* E17K mutation in ER+ breast cancer patients in the context of a clinical trial testing the AKT inhibitor AZD5363 in combination with endocrine (fulvestrant) therapy.

## Supporting information

S1 Table Overview of *AKT1* E17K reference standards. (DOCX)
