# Supplementary material for: Accurate detection of low prevalence AKT1 E17K mutation in tissue or plasma from advanced cancer patients
Source: PLoS One. 2017 May 4;12(5):e0175779. doi: 10.1371/journal.pone.0175779 (PMC5417426; doi:10.1371/journal.pone.0175779)
Supplement: S1 Table — (DOCX) [file pone.0175779.s001.docx]

**S1 Table: Overview of *AKT1* E17K reference standards**

| **Reference standard** | **Source *AKT1* E17K DNA** | **Source *AKT1* wild-type DNA** | ***AKT1* E17K mutant allele frequencies** | ***AKT1* DNA copies / mL** |
| --- | --- | --- | --- | --- |
| High quality cell line DNA | KU19-19 | human gDNA | 1%, 2%, 5%,10% | 2 x 10^5^ |
| FFPE DNA | HD167* | HD172* | 1%, 2%, 5%,10% | 2 x 10^5^ |
| ctDNA | HD658* | HD659* | 0.05%, 0.5%, 1%, 2%, 5% | 6 x 10^4^ |

* isogenic cell line pairs obtained from Horizon Diagnostics (Cambridge, UK)
